# Supplementary material for: The interactome of intact mitochondria by cross-linking mass spectrometry provides evidence for coexisting respiratory supercomplexes
Source: Mol Cell Proteomics. 2017 Dec 8;17(2):216–32. doi: 10.1074/mcp.RA117.000470 (PMC5795388; doi:10.1074/mcp.RA117.000470)
Supplement: Supplemental Data [file supp_RA117.000470_133922_0_supp_23978_fzffwf.zip › spectra_annotation/mito_DR_spectra_annotation/135-1-5-1-3-1.pdf]

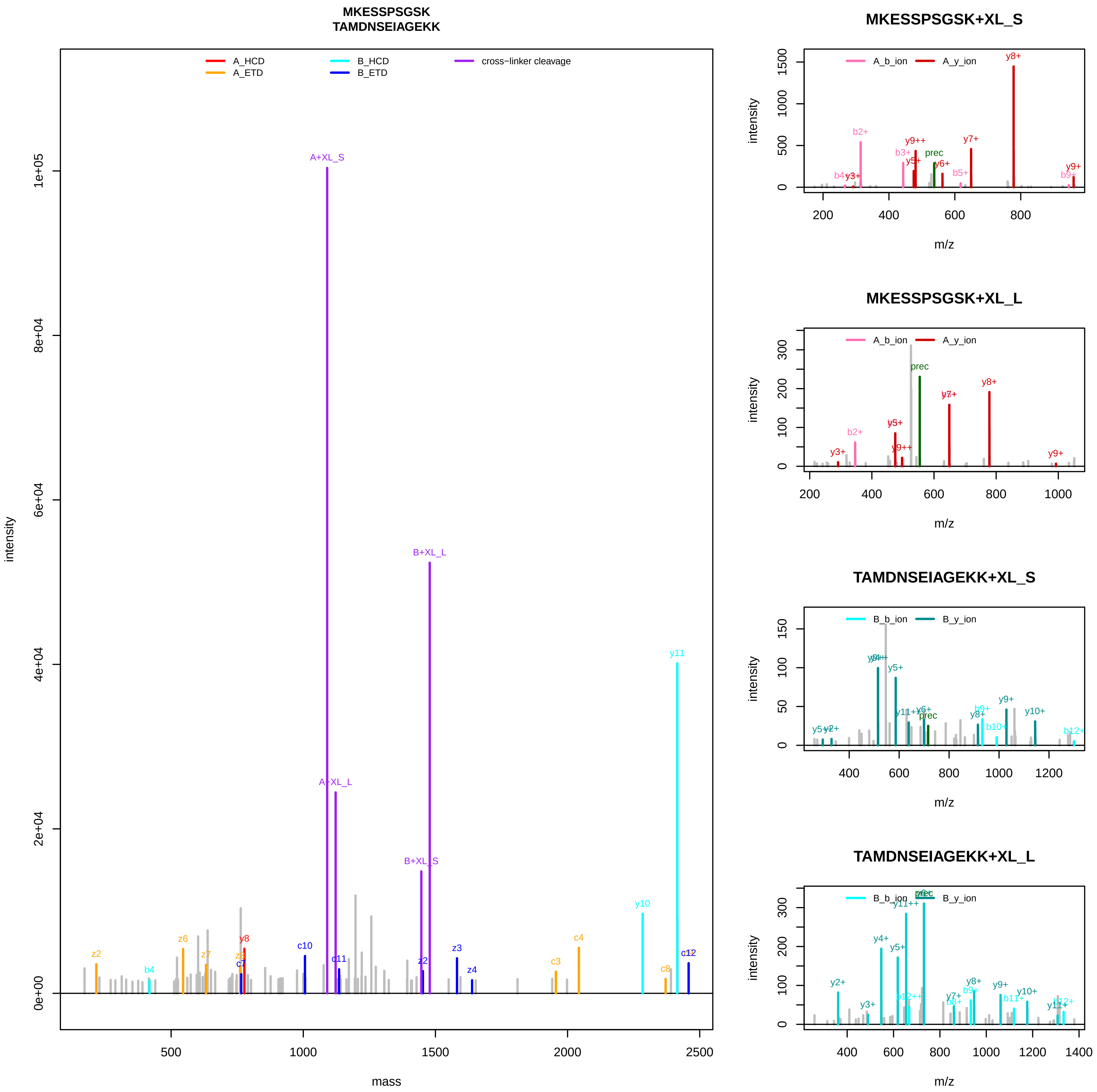
MKESSPSGSK
TAMDNSEIAGEKK
A_HCD
A_ETD
B_HCD
B_ETD
cross−linker cleavage
intensity
mass
0e+00
2e+04
4e+04
6e+04
8e+04
1e+05
500
1000
1500
2000
2500
A+XL_S
B+XL_L
A+XL_L
B+XL_S
y11
y10
z2
b4
z6
z7
y8
c7
c10
c11
z2
z3
z4
c3
c4
c8
c12
MKESSPSGSK+XL_S
MKESSPSGSK+XL_L
TAMDNSEIAGEKK+XL_S
TAMDNSEIAGEKK+XL_L
A_b_ion
A_y_ion
B_b_ion
B_y_ion
prec
m/z
y8+
y7+
y6+
y5+
y3+
y9+
y9++
b2+
b3+
b4+
b5+
b9+
y2+
y4+
y5+
y6+
y8+
y9+
y10+
y11++
b9+
b10+
b12+
y7+
y11+
b8+
b11+
y3+
b12++
